# Supplementary material for: Using the National Early Warning Score (NEWS) outside acute hospital settings: a qualitative study of staff experiences in the West of England
Source: BMJ Open. 2018 Oct 27;8(10):e022528. doi: 10.1136/bmjopen-2018-022528 (PMC6224740; doi:10.1136/bmjopen-2018-022528)
Supplement: Supplementary data [file bmjopen-2018-022528supp001.pdf]

Qualitative Evaluation of the National Early Warning Score (NEWS)- Interview Topic guide

Part A. Introduction, consent and background

- Thanks, introduce self, re-state purpose of the interview (to understand the use of NEWS in pre hospital settings)

- Discussion of how interview will be recorded, right to withdrawal, issues of confidentiality, anonymisation and informed consent. (*face-to-face written consent, telephone verbal consent).* Verbal consent: *switch audio recorder on – go through each point on written consent form.*

Part B: The use of NEWS in pre-hospital settings

- Can you tell us about your professional background and about the role in which you have encountered NEWS?
- Can you tell us about your experience of working with NEWS
    - How long have you / your organisation been using the scoring system?
    - In your organisation ➔ Who uses it? (measures, communicates, responds)
    - How do you find using it ➔ easy/difficult
    - Could you give some examples of how it is used in your organisation?
- There are 6 different clinical parameters, how do you find each of these to score?
    - **For Reference (no need to go through all of these one by one)** i) respiratory rate ii) oxygen saturations iii) temperature iv) systolic blood pressure v) pulse rate vi) level of consciousness.
    - Are all the parameters appropriate in your setting?
    - Do you calculate a score? How do you use the score ➔ are there defined responses / protocols in your organisation linked to the score?
    - Do you think that the scores and the indicated actions work/are appropriate?
    - Have the scoring levels and resulting actions caused any problems?
- How did you previously assess the severity of a patient's illness?
    - How did the NEWS tool integrate with established routines/practices in your organisation?
- How is NEWS used in your organisation
    - At 'handover of care' points?
    - As a tool to monitor clinical status in the day to day care of a patient?
    - As a tool in referral ➔ has it changed the way you / your organisation make referrals?
- Does the tool work effectively in your organisation? is it suited to your organisation?
    - How was the tool introduced?
    - Has the tool been adapted in any way to suit your particular organisation?
- What format is NEWS used - electronic or paper?
    - Is there a choice?
    - Which format would you prefer?
    - How does the format integrate with the clinical records systems that you use?
- How do other health care professionals that you work with get on with NEWS (within your organisation and / or with other organisations)?
- Have you had positive or negative experiences using the tool? Could you give examples of either of these?
- Can you think of ways in which the tool could be improved?

- Can you think of ways in which the tool could be used more effectively in your organisation and/or in other health care organisations?
- Would you like to see the tool used more widely?

Part C: Winding up

- Can you think of anyone else who it would be useful for us to talk to?
- Thanks for participating. I have covered my questions, is there anything that you would like to add?
